# A Rare Case of Cystic Prostatic Carcinoma in a Patient From Vietnam

**DOI:** 10.7759/cureus.88781

**Published:** 2025-07-25

**Authors:** Linh Tran, Hoang M Le, Dat Q Dinh, Doan H Pham

**Affiliations:** 1 Functional and Female Urology, Binh Dan Hospital, Ho Chi Minh, VNM

**Keywords:** androgen deprivation therapy, carcinoma, cystic prostatic, hematuria, hydronephrosis

## Abstract

Cystic prostatic carcinoma is a rare and aggressive variant of prostate adenocarcinoma characterized by cystic degeneration within the tumor. Due to its infrequency, clinical data regarding its presentation, optimal management, and prognosis remain limited. We report a case of a 66-year-old male who presented with a two-month history of dysuria and, more recently, hematuria. Imaging studies, including multislice computed tomography and magnetic resonance imaging, revealed a large tumor originating from the prostate, with extensive invasion into the bladder and associated bilateral hydronephrosis. Histopathological analysis confirmed cystic prostatic carcinoma, with a Gleason score of 9 (4+5) and an International Society of Urological Pathology (ISUP) grade group 5, indicating a high-grade malignancy. Given the tumor’s extensive local invasion and associated obstructive uropathy, radical surgical intervention was not feasible. A percutaneous nephrostomy was performed for palliative urinary diversion. Despite intervention, the disease progressed rapidly, and the patient succumbed to the illness six months after diagnosis.

This report underscores the aggressive nature of cystic prostatic carcinoma, which often presents with rapid progression, local invasion, and poor prognosis. Imaging and pathology are critical for distinguishing it from benign cystic lesions of the prostate, as delayed or inaccurate diagnosis can impede timely management. The presence of bilateral hydronephrosis may indicate advanced disease and has been associated with adverse outcomes in other urological cancers. Due to the scarcity of standardized treatment protocols, management is frequently individualized, and the effectiveness of androgen deprivation therapy or chemotherapy remains uncertain. Further research into molecular characterization and targeted therapies is necessary to improve outcomes for patients with this rare malignancy.

## Introduction

Cystic prostatic carcinoma is a rare and aggressive variant of prostate cancer characterized by the presence of cystic structures within the tumor mass. Unlike typical prostatic adenocarcinomas, which are predominantly solid, this subtype exhibits significant cystic degeneration, complicating both diagnosis and management [1].

The pathogenesis of cystic prostatic carcinoma is not fully understood. Some studies suggest that the cystic components may arise from ischemic necrosis within rapidly proliferating tumor tissues, leading to liquefaction and subsequent cyst formation. Others propose that these cysts result from dilated prostatic ducts obstructed by tumor growth. Additionally, certain histological variants, such as ductal adenocarcinoma, have been associated with cystic features [2].

Clinically, patients often present with lower urinary tract symptoms, including difficulty urinating, increased frequency, and acute urinary retention. In some cases, a palpable mass may be detected during a digital rectal examination. Serum prostate-specific antigen (PSA) levels are typically elevated, though exceptions have been documented [2].

Imaging studies play a crucial role in the evaluation of this malignancy. Ultrasound and computed tomography (CT) scans may reveal a large, predominantly cystic pelvic mass with irregular walls and solid nodular components. Magnetic resonance imaging (MRI) provides superior soft-tissue contrast, aiding in the detailed assessment of both cystic and solid elements of the tumor. However, definitive diagnosis often requires histopathological examination [3].

Histologically, cystic prostatic carcinoma may present as microcystic adenocarcinoma, a distinctive pattern that can appear deceptively benign at low magnification. The detection of intraluminal crystalloids or wispy blue mucin, along with immunohistochemical staining for markers such as alpha-methylacyl-CoA racemase (AMACR), can aid in accurate diagnosis [4].

Due to its rarity, there is no standardized treatment protocol for cystic prostatic carcinoma. Management strategies are typically extrapolated from those used for conventional prostatic adenocarcinomas and may include radical prostatectomy, radiation therapy, androgen deprivation therapy, or a combination thereof. The prognosis is generally poor, with a higher propensity for local invasion and distant metastasis compared to typical prostate cancers [5].

## Case presentation

A 66-year-old male with no significant past medical history presented to the urology clinic with a two-month history of progressive dysuria. The patient reported increased urinary frequency, a sensation of incomplete bladder emptying, and intermittent nocturia. There was no history of fever, flank pain, or weight loss during this period. However, one week prior to hospital admission, he noted the sudden onset of painless gross hematuria, which persisted and became more pronounced over the subsequent days.

On physical examination, the patient appeared hemodynamically stable, with no palpable abdominal masses. Digital rectal examination revealed an enlarged prostate with areas of firmness and irregularity, raising suspicion for an underlying neoplastic process. Routine laboratory tests showed mild renal impairment, with elevated serum creatinine and blood urea nitrogen levels (Table 1). Urinalysis confirmed hematuria but was negative for infection. Total prostate-specific antigen (PSA) levels were 30.26 ng/mL, with free PSA of 21.07%.

**Table 1:** Laboratory tests findings.

Laboratory tests	Value (normal range)
eGFR	64 (≥90) mL/min/1.73 m²
Serum creatinine	110 (65-120) μmol/L
Blood urea nitrogen	6.7 (2.1-8.5) mmol/L
PSA total	30.26 (<4.5) ng/mL
PSA free	21.07% (>25%)

**Figure 1:**
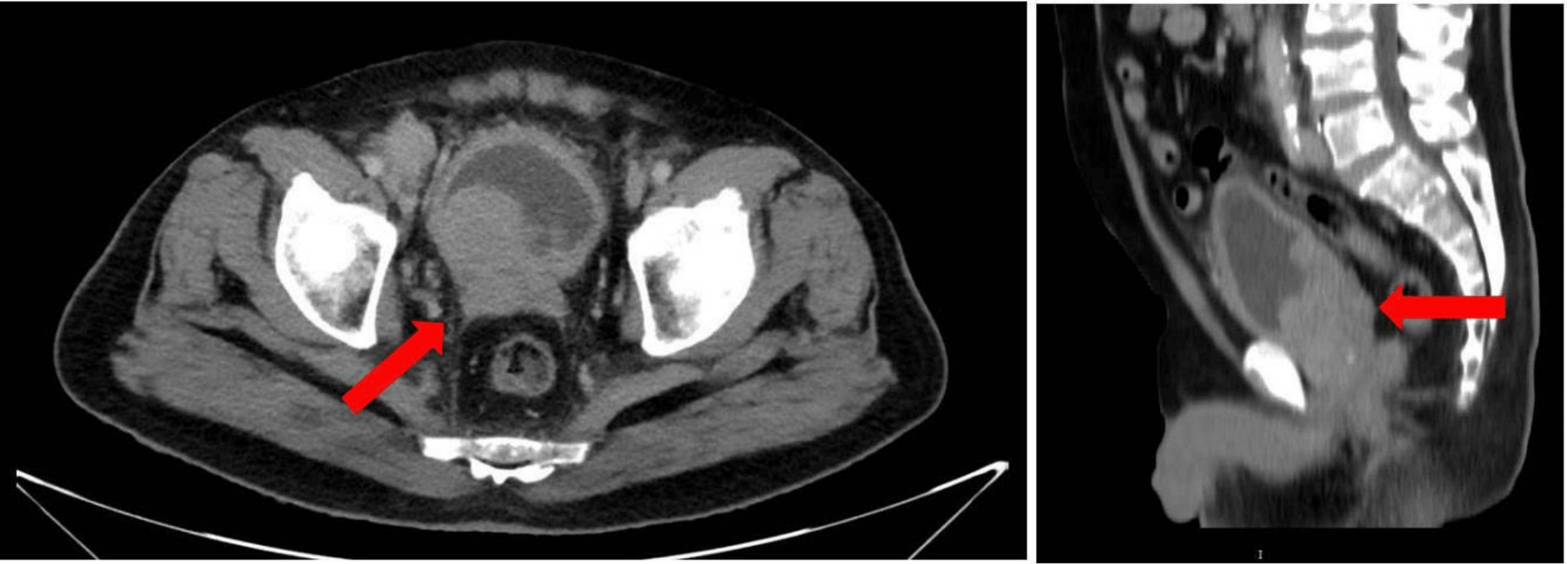
MSCT revealed cystic mass arising from the prostate, causing compression and invasion of the posterior bladder wall (arrows). MSCT: multislice computed tomography

Imaging with multislice computed tomography (MSCT) of the abdomen and pelvis revealed a large, multiloculated cystic mass arising from the prostate, causing compression and invasion of the posterior bladder wall (Figure 1). Magnetic resonance imaging (MRI) provided further delineation of the lesion, confirming extensive local invasion into the bladder and the presence of bilateral hydronephrosis, consistent with obstructive uropathy.

**Figure 2:**
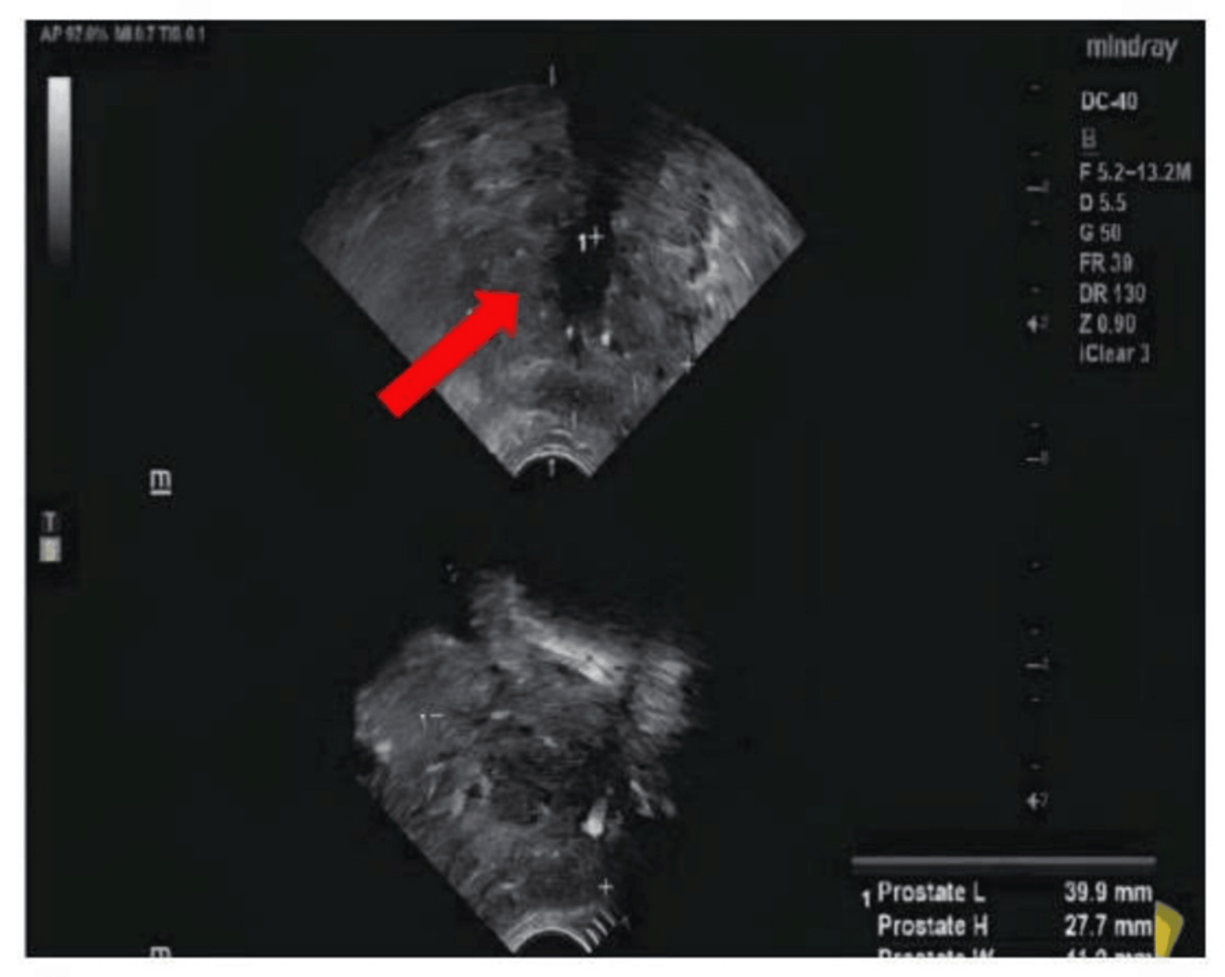
Transrectal ultrasound-guided prostate biopsy (arrow).

**Figure 3:**
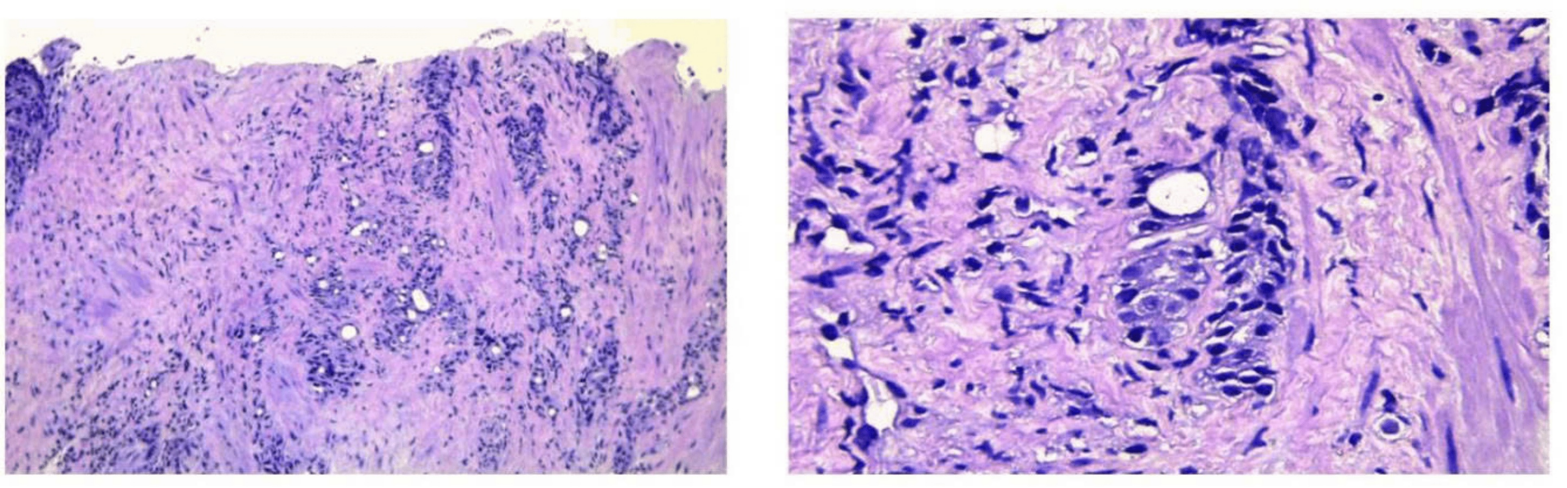
Histopathological examination revealed features consistent with cystic prostatic adenocarcinoma. The tumor exhibited a Gleason score of 9 (4+5) and was classified as ISUP grade group 5. ISUP: International Society of Urological Pathology

A transrectal ultrasound-guided prostate biopsy was performed. Histopathological examination revealed features consistent with cystic prostatic adenocarcinoma (Figure 2). The tumor exhibited a Gleason score of 9 (4+5) and was classified as International Society of Urological Pathology (ISUP) grade group 5, indicating a poorly differentiated, high-grade malignancy with aggressive behavior (Figure 3).

Given the locally advanced nature of the tumor and the extent of surrounding tissue invasion, the multidisciplinary tumor board determined that the patient was not a suitable candidate for radical prostatectomy. As an alternative, bilateral percutaneous nephrostomy was performed to alleviate the obstructive uropathy and preserve renal function. The patient was referred for palliative oncologic care. Despite these interventions, his condition continued to deteriorate due to progressive local and systemic disease. He succumbed to the illness approximately six months following the initial diagnosis.

## Discussion

Cystic prostatic carcinoma is an uncommon and aggressive variant of prostate adenocarcinoma characterized by significant cystic degeneration within the tumor mass. The presented case involves a patient diagnosed with cystic prostatic carcinoma, exhibiting symptoms of dysuria, hematuria, and bilateral hydronephrosis due to extensive tumor invasion into the bladder. This scenario underscores the complexities and challenges associated with diagnosing and managing this rare malignancy.

The patient's initial presentation with two months of dysuria followed by a week of hematuria is indicative of lower urinary tract involvement. These symptoms align with previous reports where patients with cystic prostatic carcinoma presented with similar urinary complaints. For instance, a case documented by Popov et al. described a patient with cystic prostatic carcinoma who presented with acute urinary retention and was found to have a significant prostatic cyst upon imaging [5].

Imaging findings, including MSCT and MRI, revealed extensive tumor invasion into the bladder with associated bilateral hydronephrosis. The presence of hydronephrosis suggests significant ureteral involvement and is often correlated with poor prognosis in advanced urological malignancies [5]. A study by Reis et al. demonstrated that preoperative hydronephrosis in bladder cancer patients undergoing radical cystectomy was significantly associated with advanced tumor stage and decreased overall survival [6]. While this study focused on bladder cancer, similar implications regarding hydronephrosis and prognosis may be applicable to cystic prostatic carcinoma.

Histopathological examination confirmed cystic prostatic carcinoma with a Gleason score of 9 (4+5) and an International Society of Urological Pathology (ISUP) grade group 5. These findings indicate a high-grade tumor with poor differentiation, consistent with aggressive tumor behavior. High Gleason scores have been associated with increased rates of metastasis and decreased survival in prostate cancer patients, as highlighted in a study by Humphrey [7].

The cystic nature of the tumor poses a diagnostic challenge, as it can mimic benign cystic conditions of the prostate. Previous reports emphasize the importance of correlating imaging findings with histopathological examination to avoid misdiagnosis. Popov et al. described how sonographic features of cystic prostatic carcinoma can be confused with benign cystic lesions, underlining the need for histological confirmation [5].

Due to the extensive local invasion and bilateral hydronephrosis, surgical resection of the tumor was deemed unfeasible. Instead, percutaneous nephrostomy was performed to relieve urinary obstruction and preserve renal function. This approach is commonly employed in cases of malignant ureteral obstruction when curative treatment is not an option [8]. According to Somani et al., nephrostomy can provide significant symptomatic relief and improve the quality of life in patients with advanced pelvic malignancies [9].

The role of systemic therapies such as androgen deprivation therapy (ADT) or chemotherapy in cystic prostatic carcinoma remains unclear due to its rarity. Some studies suggest that this variant of prostate cancer may not respond as effectively to standard treatments, necessitating individualized therapeutic strategies [10]. Further research is needed to determine optimal treatment modalities for this rare malignancy.

The patient's demise six months after diagnosis highlights the aggressive nature of cystic prostatic carcinoma and its poor prognosis. The presence of bilateral hydronephrosis further compounded the prognosis, as it is often a marker of advanced disease with limited treatment options [11]. A systematic review by Zhang et al. demonstrated that preoperative hydronephrosis in bladder cancer patients was significantly associated with advanced disease and decreased cancer-specific survival [12]. Although this pertains to bladder cancer, the findings may have relevance to cystic prostatic carcinoma, where hydronephrosis is indicative of extensive local progression.

Given the aggressive course of this disease, early diagnosis is crucial. However, due to its rarity, cystic prostatic carcinoma is often diagnosed at an advanced stage when curative treatment options are limited [13]. Multidisciplinary collaboration involving urologists, oncologists, and pathologists is essential to improve diagnostic accuracy and management outcomes [14].

## Conclusions

This case underscores the significant diagnostic and therapeutic challenges posed by cystic prostatic carcinoma. The disease's rarity, coupled with its aggressive behavior and poor response to conventional therapies, contributes to the unfavorable prognosis. Hydronephrosis and extensive bladder invasion, as seen in this case, further complicate treatment options. Early recognition and accurate diagnosis remain critical in managing this malignancy. However, the limited number of reported cases restricts the availability of standardized treatment protocols. Future research focusing on molecular and genetic profiling of cystic prostatic carcinoma may help identify targeted therapeutic strategies to improve patient outcomes.
